# *Cobitis takenoi* sp. n. (Cypriniformes, Cobitidae): a new spined loach from Honshu Island, Japan

**DOI:** 10.3897/zookeys.568.7733

**Published:** 2016-02-23

**Authors:** Jun Nakajima

**Affiliations:** 1 Fukuoka Institute of Health and Environmental Sciences, Mukaizano 39, Dazaifu, Fukuoka 818-0135, Japan

**Keywords:** Cobitoidei, Tango tetraploid form of *Cobitis striata*, *Cobitis* sp. 5, freshwater fish

## Abstract

A new species of spined loach, *Cobitis takenoi* **sp. n.**, is described based on the holotype and ten paratypes collected from Tango District, Honshu Island, Japan. The new species is distinguished by a combination of the following character states: 1) the lamina circularis at the base of the pectoral fin in adult male having a simple roundish plate form; 2) a narrowing of the upper segments of the first branched ray of the pectoral fin; 3) a short maxillary barbel whose length equals diameter of the eye; 4) 14 prepelvic myotomes, and 5) L3 and L5 well developed, forming longitudinal obvious stripes in males during the spawning season.

## Introduction

The genus *Cobitis* Linnaeus, 1758 (Cypriniformes: Cobitidae) includes small, slender-bodied benthic freshwater fishes. The genus is characterised by the following features: the suborbital spine is erectile; the mouth is small and inferior with three pairs of barbels; body pigmentation is organised in one dorsal and four lateral longitudinal lines or rows of blotches; and the presence of the lamina circularis at the base of the pectoral fin in adult males (Nalbant 1963, Kottelat and Freyhof 2007, Kim 2009). Approximately 80 species of the genus have been identified in Eurasia and northwestern Africa (Kottelat 2012, Nakajima 2012, Chen and Chen 2013, Chen et al. 2013, Buj et al. 2014, Erkakan and Özdemir 2014, Chen et al. 2015, Mousavi-Sabet et al. 2015, Nakajima and Suzawa 2016). Nine species within *Cobitis*, namely 1) *Cobitis biwae* Jordan & Snyder, 1901, 2) *Cobitis striata* Ikeda, 1936, 3) *Cobitis matsubarae* Okada & Ikeda, 1939, 4) *Cobitis takatsuensis* Mizuno, 1970, 5) *Cobitis shikokuensis* Suzawa, 2006, 6) *Cobitis magnostriata* Nakajima, 2012, 7) *Cobitis minamorii* Nakajima, 2012, 8) *Cobitis kaibarai* Nakajima, 2012 and 9) *Cobitis sakahoko* Nakajima & Suzawa, 2015, and six subspecies, namely 1) *Cobitis minamorii tokaiensis* Nakajima, 2012, 2) *Cobitis minamorii oumieneis* Nakajima, 2012, 3) *Cobitis minamorii yodoensis* Nakajima, 2012, 4) *Cobitis minamorii saninensis* Nakajima, 2012, 5) *Cobitis striata fuchigamii* Nakajima, 2012, and 6) *Cobitis striata hakataensis* Nakajima, 2012 have been described in Japan (Nakajima 2012, Hosoya 2013, Nakajima and Suzawa 2016).

Previously, Takeno et al. (2010) reported a *Cobitis* species from Tango District, Honshu Island, Japan, which they tentatively named as a ‘Tango tetraploid form’ of *Cobitis striata*. This species had clearly distinctive differences in body colouration patterns and mitochondrial DNA sequences as compared to other Japanese species of spined loach. Therefore, they concluded that the species was an unknown new species (Takeno et al. 2010). However, to date, this spined loach remained undescribed. In the current paper, I describe it as a new species on the basis of 11 type specimens.

## Materials and methods

**Figure 1.**
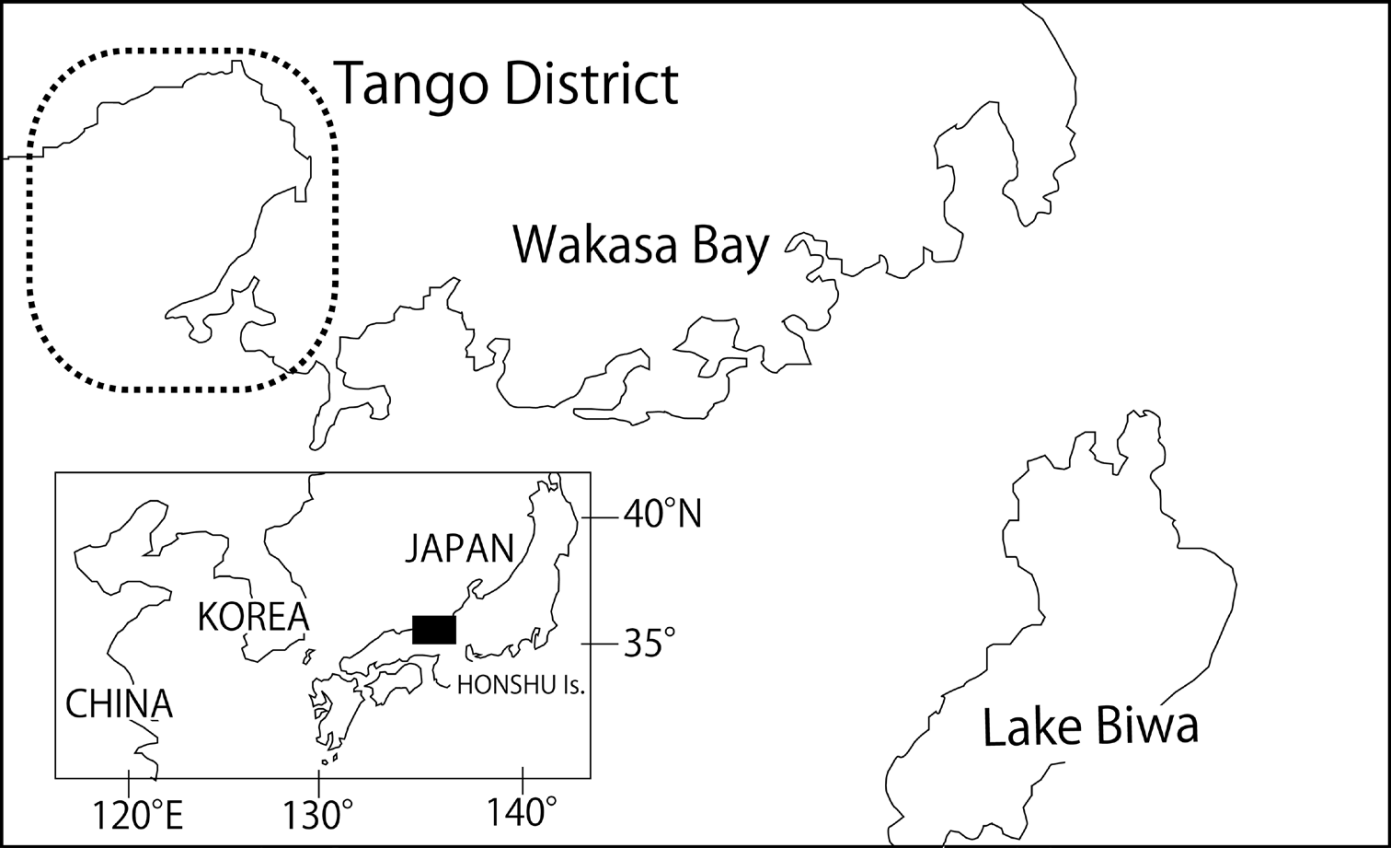
Map showing the collection area of the type series of *Cobitis takenoi* sp. n.

**Figure 2.**
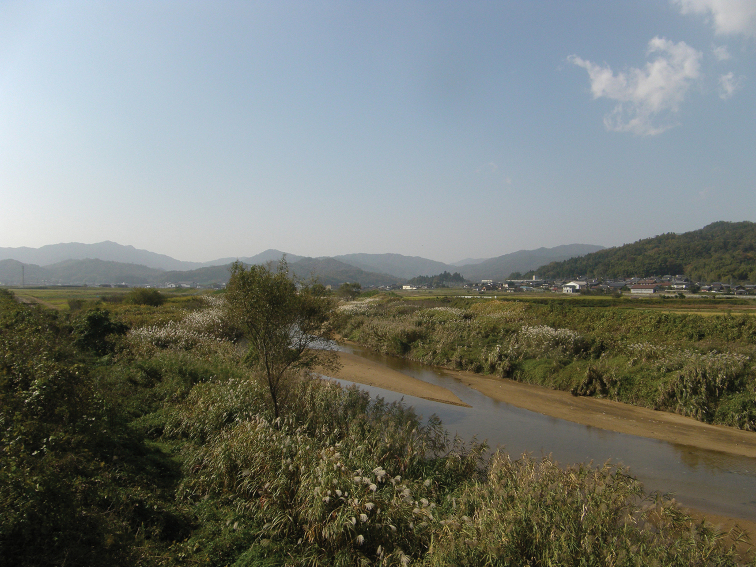
Habitat of *Cobitis takenoi* sp. n.

I examined 11 specimens collected from a small river in Tango District, Kyoto Prefecture, Honshu Island, Japan (Figs 1, 2). There is a risk of this new species being commercially overfished for the ornamental fish market (Takeno et al. 2010, Kitagawa 2015). Therefore, the precise locality of the population is not revealed in the current paper so as to protect the species. All specimens were fixed in 10% formalin and preserved in 70% ethanol. The methods used for counting and measurement of body morphological features followed Kottelat and Freyhof (2007) and Nakajima (2012). All measurements performed using a digital calliper and were recorded to the nearest 0.1 mm. The last two branched rays articulating on the last complex pterygiophore of the dorsal and anal fins were counted as one ray. The prepelvic myotome number (PMN) was defined as the number of muscle segments between the base of the pectoral fin and the origin of the pelvic fin (Nakajima 2012). The right pectoral fin of holotype and some paratypes was resected and was made transparent by placing it in 4% KOH for 24 h. After staining with alizarin red S + 1% KOH for 24 h, the lamina circularis and the upper segments of the first branched ray of the pectoral fin (USP) were observed and sketched using a stereomicroscope. The dorsal and lateral colour patterns were organised in five longitudinal lines of pigmentation, which were abbreviated as lines L1 to L5 according to the scheme of Takeda and Fujie (1945) (see also Nakajima 2012). The black spots at the caudal-fin base and the markings of the dorsal and caudal fins are additionally described.

The type series were deposited in the following collections: KPM – the Kanagawa Prefectural Museum of Natural History, Odawara, Kanagawa, Japan; TKPM – the Tokushima Prefectural Museum, Tokushima, Japan; KUN – the Faculty of Agriculture, Kinki University, Nara, Japan and JNC – private collection of the author, Japan.

## Taxonomy

### Cobitis takenoi sp. n.

Taxon classificationAnimaliaCypriniformesCobitidae

http://zoobank.org/13895F3E-53D6-4C24-BE3B-E6DBDF7BEF07

**Figure 3.**
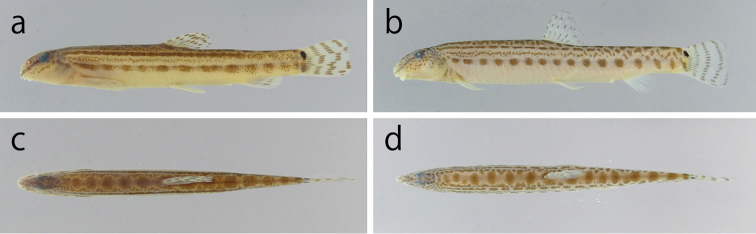
Male (**a**, **c** holotype, KPM-NI 31994, 60.4 mm SL) and female (**b**, **d** paratype, KPM-NI 31999, 70.5 mm SL) specimens of *Cobitis takenoi* sp. n. **a**, **b** Lateral view **c**, **d** dorsal view.

**Figure 4.**
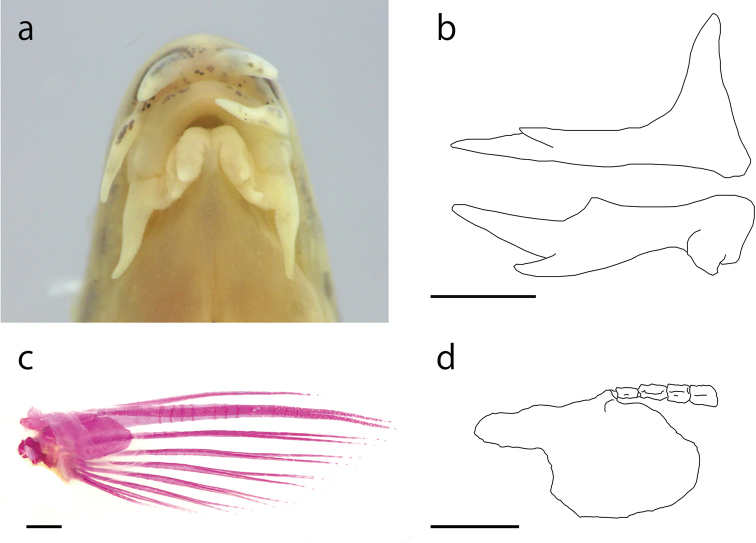
*Cobitis takenoi* sp. n., KPM-NI 31994, holotype. **a** Mouth **b** right suborbital spine, lateral view (upper), and dorsal view (lower) **c** dorsal view of the pectoral ﬁn **d** lamina circularis and upper segments of the ﬁrst branched soft ray (USP). Scale bars: 1 mm.

**Figure 5.**
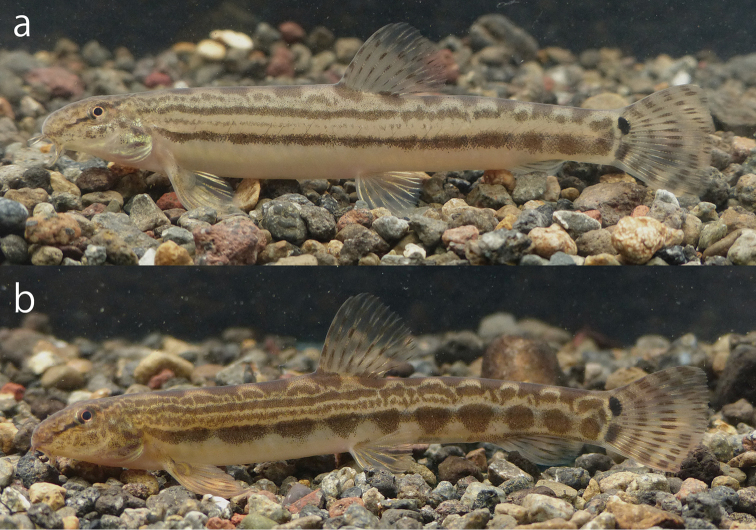
Change in colouration of the *Cobitis takenoi* sp. n. adult male (paratype, KUN-P 45133, 57.7 mm SL). **a** Spawning season, 8 July 2014 **b** non-spawning season, 5 December 2014.

Figs 3 , 4 , 5 , Table 1

Cobitis takenoi ‘Tango tetraploid form’ of Cobitis striata: Takeno et al. 2010: 108, fig. 2; Cobitis sp. 5: Nakajima et al. 2012: 92, fig. 3e; Cobitis sp.: Hosoya 2013: 331; Cobitis sp.: Kawase 2015: 181.

#### Type materials.

Holotype: KPM-NI 31994, 60.4 mm standard length (SL), male, Tango District, Kyoto Prefecture, Honshu Island, Japan; collected by K. Tominaga on 17 Apr. 2010. Paratypes: 10 specimens, all from same locality as the holotype: TKPM-P 7363, 7364, 53.2–67.5 mm SL, male and female, same data as holotype; KPM-NI 31995–31999, 49.4–70.5 mm SL, 3 males and 2 females, collected by J. Nakajima on 12 Nov. 2010; KUN-P 45133, 57.7 mm SL, male, collected by K. Tominaga on 5 Jul. 2014; JNC 188, 189, 58.6–60.6 mm SL, 2 males, same data.

#### Diagnosis.

Maxillary barbel short, more of the same eye diameter; lamina circularis in adult males simple and roundish; USP narrow; PMN 14; line L5 organised in 11–17 oblong or ovoid blotches out of spawning season, and lines L3 and L5 in adult male well-developed longitudinal obvious stripes during spawning season; upper and lower spot at caudal base not connected; tetraploid.

#### Description.

**Figure 6.**
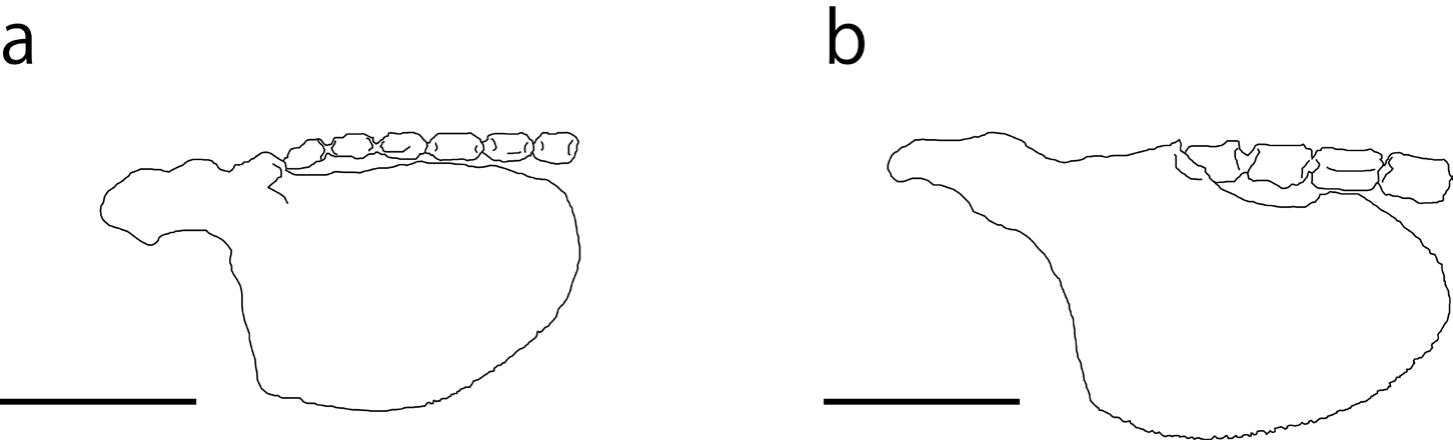
Narrow and broad types of upper segments of the first branched soft ray (USP). **a** Narrow type (*Cobitis kaibarai*) **b** broad type (*Cobitis magnostriata*). Redrawn from Nakajima (2012). Scale bars: 1 mm.

Dorsal-fin rays iii, 7; anal-fin rays iii, 5; pectoral-fin rays i, 7–8; pelvic-fin rays ii, 6; caudal-fin rays 8+8. Body elongate, laterally compressed. Head and snout elongated. Interorbital space narrow, convex. Eye relatively large. Caudal peduncle relatively compressed. Mouth small, inferior, arched with fleshy lips; lower lip divided with 2 well-developed lobes; upper lip with transverse wrinkles on the surface. Barbels, 3 pairs, first on rostorum, second on maxilla, third on maxillomandibula; each barbel well-developed, length of maxillary barbel short, same as the eye diameter; the length of the rostral and maxillary barbels shorter than that of mandibular barbel (Fig. 4a). Lateral line short, reaching the pectoral-fin base. PMN 14. Very small cycloid scales on the trunk. Suborbital spine two-pronged and incurved; length of the outer spine one-third of that of the inner spine (Fig. 4b). First branched ray of the pectoral fin longer than rest (Fig. 4c); pectoral fin in adult males longer than that in females. USP narrow (Figs 4c, d, see also Fig. 6). Lamina circularis at the base of the pectoral fin in adult males simple and roundish (Fig. 4d). Dorsal-fin base equidistant from the base of the caudal fin and the top of the snout. Pelvic-fin origin below the second or third branched dorsal fin ray. Anal fin not reaching the caudal-fin base. Margin of anal and dorsal fins slightly roundish. Caudal fin slightly roundish. Abdominal vertebrae 22 (21–23); caudal vertebrae 20 (19–21); total vertebrae 42 (40–44) (Takeno et al. 2010). Largest recorded specimens 65.5 mm SL in male and 84.9 mm SL in female (Takeno et al. 2010).

**Table 1.** Counts and morphometric measurements of *Cobitis takenoi* sp. n.

		Holotype	Paratypes	
			7 males	3 females
**SL (mm); mean (range)**		60.6	55.5 (49.4–58.6)	67.4 (64.2–70.5)
**Counts**	Dorsal fin	iii, 7	iii, 7	iii, 7
	Anal fin	iii, 5	iii, 5	iii, 5
	Pectoral fin	i, 8	i, 7–8	i, 7–8
	Pelvic fin	ii, 6	ii, 6	ii, 6
	Caudal fin	8+8	8+8	8+8
**In % SL; mean (range)**	HL	20.0	20.7 (19.8–21.7)	20.2 (19.3–21.5)
	Body depth	15.7	14.4 (13.2–17.6)	13.3 (12.5–13.9)
	Predorsal length	50.2	49.7 (47.6–53.1)	51.1 (49.5–52.6)
	Preanal length	74.6	74.8 (71.2–77.1)	76.0 (73.9–78.8)
	LPP	32.0	31.6 (28.6–33.6)	34.0 (32.2–35.4)
	LPA	25.7	25.2 (24.2–26.9)	25.7 (24.9–27.3)
	DCP	9.7	9.5 (9.0–10.8)	9.3 (8.8–9.6)
**In % HL; mean (range)**	Snout length	35.9	36.4 (31.6–44.2)	43.0 (42.3–44.2)
	Eye diameter	18.8	19.7 (17.9–21.9)	17.9 (17.7–18.1)
**PMN**		14	14.0	14.0

SL , standard length ; HL , lateral head length ; LPP , length of between pectoral-ﬁn base and pelvic-ﬁn origin ; LPA , length of between pelvic-ﬁn base and anal-ﬁn origin ; DCP , depth of caudal peduncle ; PMN , prepelvic myotome number

#### Colouration.

Body yellowish white with dark brown pigmentation in fresh. A clear streak running from the tip of the snout to the occiput, crossing to the eye. Upper part of the head covered with amorphous spots; opercle and snout covered with amorphous patterns. Caudal and dorsal fins with 3–4 arcuate bars. Anal fin pigmented along fin rays. Upper spot at caudal base jet-black, size comparable to the eye diameter, lower spot at the caudal base relatively inconspicuous and small; upper and lower spots at the caudal base not connected. ***Male out of spawning season*** (Figs 3a, 5b). Body pigmentation organised in 1 dorsal and 4 lateral lines. Line L1 consisting of a series of 11–16, saddle or oval shaped blotches. Line L2 formed by a longitudinal jugged line or convex semicircular spots or chained small angular blotches, only present on dorsal part of body. Line L3 formed by a sharp longitudinal line or narrow dotted line, reaching to the post-dorsal body, with intermissive posterior part. Line L4 formed by narrow web like line or dots, reaching to dorsal body. Line L5 organised in 11–17 blotches from the upper part of the pectoral fin to the caudal-fin base; blotches roundish, frequently oblong or ovoid. ***Male in the spawning season*** (Fig. 5a). Line L4 not visible or formed by faint longitudinal line. Lines L3 and L5 well developed, forming longitudinal obvious stripes from the upper part of the pectoral-fin base to the caudal-fin base, often intermissive posterior part of L3. ***Female*** (Fig. 3b). Similar to males out of spawning season.

#### Sexual dimorphism.

Males having a roundish lamina circularis at the base of the pectoral fins; females do not. Generally, the body size of females larger than that of males. Lines L3 and L5 of adult males well developed, forming longitudinal obvious stripes during the spawning season; females do not.

#### Ploidy.

Tetraploid (Takeno et al. 2010).

#### Etymology.

The specific name is dedicated to Mr. Makoto Takeno, the discoverer of this spined loach.

#### Distribution.

Tango District, Kyoto prefecture, Honshu Island, Japan.

#### Habitat and biology.

This species inhabits sandy-mud bottoms of the middle and lower reaches of rivers (Fig. 2). Life histories are unknown.

#### mtDNA cyt*b* sequence.

AB533231–AB533234 (Takeno et al. 2010).

#### Japanese name.

Tango-suji-shima-dojyô (Nakajima et al. 2012).

#### Comparison.

This new species is distinguished from nine species of *Cobitis* in the Japanese archipelago (*Cobitis biwae*, *Cobitis striata*, *Cobitis matsubarae*, *Cobitis takatsuensis*, *Cobitis shikokuensis*, *Cobitis magnostriata*, *Cobitis minamorii*, *Cobitis kaibarai* and *Cobitis sakahoko*) by a combination of the following character states: a short maxillary barbel equaling in length the eye diameter (vs. longer than the eye diameter in *Cobitis matsubarae*, *Cobitis takatsuensis*, *Cobitis shikokuensis* and *Cobitis sakahoko*); a simple roundish lamina circularis (vs. beak-shaped or narrow in *Cobitis biwae*; quite narrow in *Cobitis takatsuensis* and *Cobitis shikokuensis*; rectangular with a neck in *Cobitis sakahoko*); a narrow USP (vs. broad in *Cobitis matsubarae*, *Cobitis takatsuensis*, *Cobitis shikokuensis*, *Cobitis magnostriata* and *Cobitis sakahoko*); PMN 14 (vs. commonly 12 in *Cobitis minamorii*; commonly 13 in *Cobitis striata* and *Cobitis kaibarai*); a L5 formed of blotches out of spawning season (vs. stripe-like in and out of spawning season in *Cobitis takatsuensis* and *Cobitis magnostriata*); both spots at caudal base obvious (vs. lower spot inconspicuous in *Cobitis striata* and *Cobitis kaibarai*); and ploidy tetraploid (vs. diploid in *Cobitis striata*, *Cobitis takatsuensis*, *Cobitis shikokuensis*, *Cobitis minamorii* and *Cobitis kaibarai*). These comparative data were summarised from Nakajima and Suzawa (2016).

#### Remarks.

Till date, *Cobitis takenoi* has only been found in one small river system, and the habitat is under threat from river improvement. In addition, some threatened freshwater fishes are captured and sold illegally in Japan (e.g. *Parabotia curtus*, Watanabe et al. 2015), and this new species is similarly at the risk of being commercially overfished for the ornamental fish market (Takeno et al. 2010). Therefore, the species is ranked as a critically endangered species (CR) – as *Cobitis* sp. – in the Japanese Red List (Kitagawa 2015). The distribution pattern, suitable habitat and life history of this species are not well-known. Basic biological investigations are required for its effective conservation.

## Supplementary Material

XML Treatment for Cobitis takenoi
